# Implementation of Continuous Glucose Monitoring in the Hospital: Emergent Considerations for Remote Glucose Monitoring During the COVID-19 Pandemic

**DOI:** 10.1177/1932296820932903

**Published:** 2020-06-14

**Authors:** Rodolfo J. Galindo, Grazia Aleppo, David C. Klonoff, Elias K. Spanakis, Shivani Agarwal, Priya Vellanki, Darin E. Olson, Guillermo E. Umpierrez, Georgia M. Davis, Francisco J. Pasquel

**Affiliations:** 1 Division of Endocrinology, Metabolism and Lipids, Department of Medicine, Emory University School of Medicine, Atlanta, GA, USA; 2 Division of Endocrinology, Metabolism and Molecular Medicine, Feinberg School of Medicine, Northwestern University, Chicago, IL, USA; 3 Diabetes Research Institute, Mills-Peninsula Medical Center, San Mateo, CA, USA; 4 Division of Endocrinology, Baltimore Veterans Affairs Medical Center, MD, USA; 5 Division of Endocrinology, Diabetes, and Nutrition, University of Maryland School of Medicine, Baltimore, USA; 6 Fleischer Institute for Diabetes and Metabolism, NY-Regional Center for Diabetes Translational Research, Albert Einstein College of Medicine, Bronx, NY, USA; 7 Division of Endocrinology, Atlanta Veterans Affairs Medical Center, GA, USA

**Keywords:** diabetes mellitus, type 2, CGM, COVID-19, inpatient, hospitalized, continuous glucose monitoring

## Abstract

Continuous glucose monitoring (CGM) has become a widely used tool in the ambulatory setting for monitoring glucose levels, as well as detecting uncontrolled hyperglycemia, hypoglycemia, and glycemic variability. The accuracy of some CGM systems has recently improved to the point of manufacture with factory calibration and Food and Drug Administration clearance for nonadjunctive use to dose insulin. In this commentary, we analyze the answers to six questions about what is needed to bring CGM into the hospital as a reliable, safe, and effective tool. The evidence to date indicates that CGM offers promise as an effective tool for monitoring hospitalized patients. During the current coronavirus disease 2019 crisis, we hope to provide guidance to healthcare professionals, who are seeking to reduce exposure to SARS-Cov-2, as well as preserve invaluable personal protective equipment. In this commentary, we address who, what, where, when, why, and how CGM can be adopted for inpatient use.

## Introduction

The incredible pressure forced upon healthcare systems worldwide by the coronavirus disease 2019 (COVID-19) pandemic has led to a crisis situation. Emergent changes in hospital patient care have been made to address critical supply shortages, most notably the lack of personal protective equipment (PPE) available to healthcare workers (HCW).^1,2^ The increasing proportion of hospitalized patients with diabetes mellitus (DM) and concurrent COVID-19 only increases the burden on healthcare systems, forcing HCW to choose between providing necessary bedside care and maintaining their own personal safety in light of extreme limitations in available PPE.

Three main driving forces relate to this crisis in inpatient diabetes care, including (1) the large number of hospitalized patients with diabetes and COVID-19, (2) burned-out HCW, and (3) a lack of resources. These circumstances have raised concerns regarding the most appropriate way to address glycemic control during this public health crisis. We know that appropriate glycemic control may improve patient-centered outcomes by reducing hospital complications and length of stay. However, an approach is needed that also addresses priorities of community-centered care, such as preserving supplies of PPE, minimizing exposure risk to HCW, and limiting transmission to the community.^1-3^ The traditional approach to care for patients with diabetes in the hospital is complex and requires portable glucose monitors for frequent point-of-care (POC) testing with fingersticks and associated technical and comfort limitations. Four or more capillary blood glucose measurements per day are typically recommended for patients receiving multiple daily insulin injections, with hourly glucose measurements recommended for those receiving intravenous continuous insulin infusion (i.e., patients with critical illness, diabetic ketoacidosis, or hyperosmolar hyperglycemic state). These recommendations have been rendered nearly unachievable under current conditions, where an increased need for bedside glucose monitoring has been undermined by rising patient-to-nurse ratios, an increase in the overall acuity of the inpatient population, and a scarcity of PPE. These consequences have prompted HCW to change standard practice to reduce bedside encounters.^1^ Continuous glucose monitoring (CGM) is one of the technologies being implemented during this ongoing transformation of care. Factory-calibrated CGM provides a uniquely practical alternative, by allowing real-time glucose monitoring and alarms to prevent untoward glycemic issues, while reducing the need for frequent inpatient fingerstick testing. Less frequent fingerstick testing, in turn, decreases exposure time and PPE use.

In early April 2020, the Food and Drug Administration (FDA) did not object to the use of CGM devices in the inpatient setting in response to the COVID-19 pandemic.^1^ Abbott and Dexcom offered to supply hospitals with CGM systems to help monitor glucose levels remotely and are helping hospital clients learn how to use these systems.^4,5^ The appropriate implementation of CGM may help decrease the burden of inpatient diabetes care. It is not known, however, whether hospitals utilizing real-time CGM during this pandemic uniformly have the resources and expertise necessary for effective implementation, including the ability to provide appropriate training to HCW.^4,5^

Anecdotal reports describe widespread use of CGM in many US hospitals during this pandemic. However, unknown costs related to implementation and lack of clinical outcome data necessitate careful patient selection to: (1) optimize potential glycemic benefits that could be achieved by using CGM and (2) preserve the supply of CGM devices.^1,6^ Hence, there is a need for appropriate patient selection; sufficient staff training; and acceptance from clinicians, nursing staff, and hospital administration. In this commentary, we hope to provide practical advice to HCW trying to implement this technology in the hospital to preserve critical resources (PPE), reduce nursing burden, and reduce exposure risk. Our recommendations are based on our clinical and recent research experience with CGM in the hospital setting,^7-10^ which includes ongoing clinical trials (NCT03508934 and NCT03877068).

### Who Should Use CGM in the Hospital?

Considerations about individualization of therapy may help identify patients most likely to benefit from CGM.^1^ Patients admitted with mild-to-moderate hyperglycemia (i.e., blood glucose <180-200mg/dL), and not on outpatient insulin therapy, may be effectively treated with simple regimens requiring less frequent glucose testing.^1,11^ However, patients with moderate to severe hyperglycemia (i.e., blood glucose ≥200mg/dL) require treatment with more complex insulin regimens after they arrive in the hospital. Such patients include those with: (1) type 1 DM; (2) regimens of high-dose insulin or multiple noninsulin agents; (3) a long-standing history of diabetes; (4) significant glucotoxicity related to the current infection, new DM diagnosis, or chronically uncontrolled hyperglycemia; or (5) iatrogenic hyperglycemia caused by high-dose steroid or medical nutrition therapy (enteral or parenteral nutrition). Anecdotally, patients with severe COVID-19 can be very insulin resistant on presentation and require high doses of insulin (personal report from SA), a phenomenon that has also been observed in the United Kingdom.^12^ These groups of patients will require frequent glucose monitoring that could potentially be accomplished with CGM. In addition to patients on complex insulin regimens, CGM initiation may be prioritized in frail patients, or those with comorbidities increasing the risk of hypoglycemia (i.e., poor nutrition, renal failure, or advanced age).^1^

Patients already using CGM in the outpatient setting may continue using their personal devices in the hospital.^13^ Many institutions have already established standard/institutional protocols for self-use of CGM in hospitalized patients. Patients without adequate CGM self-management skills could be assisted with inpatient use through consultation with diabetes technology experts (e.g., physician specialists, diabetes nurse champions, or certified diabetes educators). Remote CGM education and training available for outpatients, provided by institutional diabetes educators or CGM manufacturers, will probably be very helpful for staff and patients in the hospital. Current training programs provided by each manufacturer are simple, short, and easy to understand.

In addition, one feature of an ideal CGM candidate may be having a smartphone (personal or from the facility) that can relay information from the CGM inside the patient’s room to any device with internet capability outside of the room. For patients wearing the Abbott FreeStyle Libre device (Abbott Diabetes Care Inc., Alameda, CA, USA), it would be expected that they are able to actively participate and scan (“flash”) the device intermittently. Many HCW are currently knowledgeable or can quickly learn how to apply the technology, as well as receive and respond to the data.

### What CGM Devices are Available?

Currently available subcutaneous (SC) CGM devices in the United States include two classes: those that are factory-calibrated and those that are calibrated by the patient. Factory-calibrated CGM (not requiring calibration with capillary glucose) includes Abbott FreeStyle Libre 14 day Flash Glucose Monitoring System and Dexcom G6 (Dexcom, Inc., San Diego, CA, USA). Devices that require intermittent calibration include: Medtronic Guardian 3 and Enlite 2 (both by Medtronic, Inc., Northridge, CA, USA) and Eversense (Senseonics, Inc., Germantown, MD, USA)—the last one is the only FDA-cleared implantable CGM.^14,15^ Intravascular devices, with potentially acceptable accuracy, have been tested in critically ill patients, but are not widely used.^16^ No consistent benefit has been observed with the use of CGM devices in the intensive care unit (ICU); however, currently reported outcome data is derived from small underpowered studies. The two CGMs being delivered to hospitals during the current pandemic are the FreeStyle Libre and G6, which are both factory-calibrated, thereby reducing the need for capillary glucose monitoring (Table 1). Despite benefits supported by a large body of evidence in ambulatory patients, evidence for the use of these CGM systems in hospitalized patients is limited.^6^

**Table 1.** Factory-Calibrated CGM Devices for Patients Admitted to the Hospital With COVID-19.

	Dexcom G6	Abbott FreeStyle Libre
Warm up	• 2 h	• 1 h (recommend POC during first 12 h if therapy changes are considered)
BG range	• 40-400 mg/dL “LOW” = <40 mg/dL “HIGH” = >400 mg/dL	• 40-500 mg/dL “LO” = <40 mg/dL “HI” = >500 mg/dL
Duration	• 10 d	• 14 d
Potential interactions	High doses acetaminophen (>1000 mg/q 6 h), hydroxyurea	Aspirin (high doses), Vitamin C (high doses > 1-2 g/d)
Potential limitations	• Lag time, particularly during times of rapid glucose changes • Over-reacting to trend arrows, and risk of extra insulin dosing and stacking • Sensor failure/malfunctioning • Prone ventilation practice for • COVID-19 patients, limiting abdomen site placement	Lag time, particularly during times of rapid glucose changes • Over-reacting to trend arrows, and risk of extra insulin dosing and stacking • Sensor failure/malfunctioning • Need for sensor flashing at least every eight hours (ideally by the patient)
App for the patient’s device (compatibility)	• Dexcom G6 app (iOS or Android OS)	• FreeStyle LibreLink app (iOS or Android OS)
App for the “follower’s” device (compatibility)	• Dexcom Follow app (iOS or Android OS)	• LibreLinkUp app (iOS or Android OS)
Software to review glucose profiles (Note: there is a two to three hours data delay for both systems)	• https://clarity.dexcom.com • Glooko (Diasend)	• https://www.libreview.com/ • Glooko (Diasend)
Ongoing inpatient interventional studies	• Dexcom intervention trial (NCT03877068) • CGM in Hospitalized Veterans (NCT03508934) • Scripps Digital Diabetes (NCT04269655) • Green Line From Hospital to Territory (GreenLightHT) (NCT03764709) • Use of Wearables for Early Detection of Complications After Major Acute Abdominal Surgery (NCT04257344)	• DRIVE—Perioperative Period (DRIVE-Periop) (NCT04033705) • Flash Glucose Measurement in Patients on Total Parenteral Nutrition (NCT03871660) • Early Glargine (Lantus) in DKA Management in Children With Type 1 Diabetes (NCT03107208)
Consider prioritizing	• Multiple daily injections • Frail patients or those with poor nutrition, renal failure, or advanced age • High glycemic variability • Steroid-induced hyperglycemia, enteral/parenteral-induced hyperglycemia • Type 1 diabetes	
Glucose confirmation by POC	• Hypoglycemia symptoms without corresponding readings • Hypo- or hyperglycemia values without symptoms • Glucose changing rapidly (>2 mg/dL/min) • No current glucose reading or glucose trend arrow • When you see the blood drop symbol, you must check your blood glucose • Glucose values <85 or >300 mg/dL • For first 24 h of wear • Perioperative period	
ICU	• If appropriate, guide therapy by glucose POC testing (q2-q4hr while on IV insulin (no experimental data available) • Transition to SC insulin if clinically appropriate	

Abbreviations: BG, blood glucose; CGM continuous glucose monitoring; COVID-19, coronavirus disease 2019; DKA, diabetic ketoacidosis; ICU, intensive care unit; MRI, magnetic resonance imaging; POC, point of care; SC, subcutaneous.

The costs required for each device should be considered. For example, the FreeStyle Libre can require the purchase of only a sensor for patients who already have a compatible smartphone. A reader or another smartphone from the facility would need to be supplied to other patients. The G6 requires both a sensor, and a relatively expensive transmitter, as well as a compatible smartphone or reader. Hospitals should make plans ahead of time about whether the transmitters and readers will become the property of the patient or be sterilized and used for additional patients. No official information is available regarding potential sterilization and reuse of these devices during this pandemic. Even when data can be transmitted outside the room, HCW must be ready to receive and use the CGM data. Getting data to HCW can be as simple as using a personal smartphone in a provider’s pocket if using a “follower” App (see section How to Implement CGM into the Hospital?), but some systems may have Health Insurance Portability and Accountability Act (HIPAA) concerns, and some HCW may not be willing to incur this responsibility. In addition, multiple providers care for patients with COVID-19. Doctors and nurses have different shift schedules, and considerations about who receives the data among subsequent shifts need to be delineated to avoid gaps in care.

### Where can CGM be Implemented?

There is a vast body of literature citing studies of CGM in the ICU; however, small sample sizes, heterogeneity in design, and prespecified nonclinical outcomes in most of these studies preclude strong recommendations for use.^13^ The current recommended target glucose range in critically ill patients is 140-180 mg/dL.^17^ It is difficult to achieve this range with multiple daily SC insulin injections, particularly in patients receiving vasoactive agents, high doses of steroids, or medical nutrition therapy (MNT). Computerized algorithms are effective to treat such patients, but typically require frequent input of capillary glucose values to adjust insulin therapy.^6^ A detailed review of CGM devices in the inpatient setting (ICU and non-ICU) and computerized algorithms was recently presented by Davis et al.^6^

There are limited data about the performance of current-generation SC CGMs in sicker patients (e.g., with shock, anasarca, and end-stage renal disease on hemodialysis or peritoneal dialysis).^6^ Intravenous CGM devices may provide higher accuracy than SC devices, particularly during surgery, but implementation is more time consuming and complex.^18^ The FreeStyle Libre may overestimate lower glucose levels in both ICU and non-ICU patients,^9,10,18^ particularly in the perioperative period.^18^ Similar findings have been observed in patients undergoing coronary artery bypass graft surgery with the G6 (FJP, personal communication). Hence, we do not recommend the use of CGM to guide therapy in the operating room or immediate postoperative period. Placement of a new sensor is recommended after surgery.

A small study (*n* = 8) involving ICU patients with diabetes compared the accuracy of the FreeStyle Libre CGM with capillary or arterial blood glucose.^19^ The overall mean absolute relative difference (MARD) was ~14% compared to 185 arterial glucose samples and ~20% compared to 89 capillary samples. There were no statistically significant accuracy differences in the presence of vasopressor therapy. Accuracy studies with the G6 in ICU patients are ongoing. The performance of these two types of sensors during hypotension or hypoxia has not been reported. To our knowledge, there are no clinical trials evaluating relevant clinical outcomes with the use of CGM in the ICU setting.

Data from non-ICU medical or surgical wards are limited.^6^ Compared to measuring capillary POC glucose concentrations before meals and bedtime, there are theoretical considerations, derived from small observational studies, that suggest CGM use in the non-ICU wards may detect and allow prevention of hypoglycemia.^6^ This potential benefit is particularly likely for asymptomatic and/or nocturnal hypoglycemia, as well as severe hyperglycemic episodes.^13^ Recent clinical trials are focusing on the evaluation of currently available factory-calibrated sensors, such as the G6 and FreeStyle Libre. Among insulin-treated hospitalized patients with type 2 diabetes, the FreeStyle Libre-Pro (with blinded results) showed lower performance for glucose levels <80 mg/dL compared to higher values.^9^ It should be recognized going forward that the process for monitoring CGM quality standards in the hospital may need to be adapted. Clinical Laboratory Improvement Amendments requirements assume the presence of a physiologic sample (e.g., blood) for processing, which is unavailable with continuous monitoring of interstitial glucose.

Ongoing efforts from Spanakis et al have shown that the implementation of a glucose telemetry system (GTS)^20^ is feasible. This system is currently being evaluated in clinical trials enrolling hospitalized patients including those at risk for hypoglycemia (see section How to Implement CGM into the Hospital?).

### When Should We Be Concerned About Biological, Imaging, or Other Interferences?

Several molecules may cause interference in glucose readings from the two factory-calibrated CGMs (Table 1). These systems contain the enzyme glucose oxidase, which is affected by various interferents. The FreeStyle Libre uses a wired glucose oxidase-based enzyme technology with redox sensing membrane,^21^ which reduces susceptibility to extreme oxygen levels (both low or high), and minimizes the response to electrochemical interfering substances at therapeutic or physiologic levels, such as acetaminophen and uric acid.^22^ Ascorbic acid may falsely elevate sensor glucose readings, while aspirin may slightly lower glucose readings with FreeStyle Libre. The G6 system uses an “advanced” (proprietary) version of electrochemical glucose oxidase-based technology, with a semi-permeable membrane coating. In a pilot study by Basu et al testing the susceptibility of previous generation Dexcom and Medtronic CGM sensors (G4 Platinum and Medtronic Guardian Soft-Sensor), it was shown that several substances may interfere with reading of interstitial glucose by CGM sensors, including: acetaminophen, ethanol, albuterol, lisinopril, atenolol, atorvastatin, and wine.^21,23^ In the latest version (G6), the addition of a permselective membrane coating to the sensor surface inhibits the diffusion of molecules that can generate potentially spurious signals into the sensor, eliminating the risk of acetaminophen interference at therapeutic doses (up to 1000 mg every six hours). There is no interference with this drug in the FreeStyle Libre.^24^ A recently recognized interference by hydroxyurea has also been noted with the G6, and the manufacturer has recommended discontinuation of the CGM in patients using this medication.

For imaging procedures consider placing a lead apron over the sensor for X-rays, computed tomography (CT) imaging, angiogram, or fluoroscopy. Sensors must be removed for magnetic resonance imaging (MRI), and a new sensor may be needed after completion of the study. CGM sensors need not be removed for ultrasound imaging (Table 1). Migdal et al recently evaluated the potential interference of radiological procedures (X-rays, CT scan, or angiography) with the G6 among hospitalized patients. No significant changes in mean sensor glucose values, MARD, or glycemic variability (as measured by coefficient of variation) were observed with this device after imaging procedures. Subjects undergoing MRI were excluded, given concerns about the potential impact of magnetic fields on CGM function and safety.^25^ Recently, Thomas et al exposed G6 sensor/transmitter pairs to X-rays to simulate a radiotherapeutic procedure and to radiofrequency and magnetic fields to simulate diagnostic MRI.^26^ Consistent with the clinical observations,^25^ the devices appear unlikely to be affected by X-irradiation used in imaging studies, except during MRI.^26^ Simulated MRI conditions, however, created displacement force, minimal heating, and current in sensor/transmitter pairs.^26^

There is a potential impact on performance during surgical conditions. Confirmation of CGM accuracy by comparison with a POC glucose measurement is recommended to ensure sensor accuracy is preserved after surgery. Schierenbeck et al reported a small study (*n* = 24) comparing the accuracy of the Eirus Intravascular (Maquet Critical Care AB, Solna, Sweden), which is not FDA cleared, vs FreeStyle Libre CGM among patients undergoing cardiac surgery. Both systems use the glucose oxidase method.^18^ The authors reported no problems with the FreeStyle Libre regarding insertion or data gap, with only one sensor detaching because of excessive sweating, for the duration of the monitoring study (mean 47 ± 7 hours). However, the FreeStyle Libre CGM underestimated glucose levels (Eirus vs FreeStyle Libre, mean glucose 147 ± 31 vs 102 ± 32 mg/dL), with an overall MARD (matched CGM-reference arterial glucose pairs: 578) of 30.5% ± 12.4%, with the MARD of individual sensors ranging from 12% to 52%. An ongoing ancillary study is evaluating stress hyperglycemia with the G6 during cardiac surgery (NCT03743025). In a recent study comparing the FreeStyle Libre Pro with POC including noncritically ill patients with type 2 diabetes, the overall MARD was 14.8% (ranging between 11.4% and 16.7%) for glucoses between 70 and 250 mg/dL. Clark Error grid analysis showed 98.0% of glucose pairs within Zones A and B (1829 POC-CGM matched glucose pairs). The CGM accuracy was lower for glucose values <70 mg/dL.^10^

There are other factors to consider that may impact CGM accuracy and reliability. Reports of infections, skin reactions, or open wounds are rare. Some patients may have an allergy to isobornyl acrylate-based adhesive, used with the FreeStyle Libre CGM and Omnipod insulin pump (Insulet Corporation, Acton, MA), for which the G6 provides an alternative.^27^ The nonlinearity of CGM devices outside of the typical target glucose range (70-180 mg/dL) should be considered when patients approach more optimal glucose levels, as CGM devices may “overcall” hypoglycemia. While we await more data from randomized controlled trials (RCTs), among patients approaching hypoglycemia, POC glucose confirmation is recommended. This may be of specific importance in COVID-19, where many patients are reported to have significant glucotoxicity that can often resolve rapidly with increased insulin sensitivity and increased risk of iatrogenic hypoglycemia. If hypoglycemia is confirmed, then hospital hypoglycemia protocols should be followed including a repeat POC glucose test at 15 minutes to ensure correction.

### Why Should We Test POC Capillary Blood Glucose in Patients Using CGM?

While we learn more about CGM use in the hospital a hybrid model utilizing both POC testing and CGM (POC + CGM) may be indicated in certain situations to ensure readings are valid and opportune. Even though these devices are factory-calibrated, several potential scenarios in the hospital (e.g., interfering substances, MRI, hypoxia, acidosis, and hypotension) require careful use of this technology in such patients. We recommend avoiding CGM use in patients with skin infections, anasarca, and those treated with vasoactive agents (Table 2). We recommend checking POC capillary glucose in situations of rapid glucose changes (>2 mg/dL/min), outside of a predetermined range (i.e., glucose levels <85 or >300 mg/dL), or when patient symptoms do not correlate with CGM readings. For the FreeStyle Libre, POC glucose measurements are recommended any time a magnifying glass appears on the display device (e.g., for the first 12 hours, rapid change, and hypoglycemia). For the G6, POC glucose measurement is required during the two-hour warm-up period or if there is a number without a corresponding trend arrow to facilitate making insulin dose decisions (Table 1).

**Table 2.** Clinical Considerations During the Current COVID-19 Pandemic in the Setting of Emergent Changes in Practice Due to Limited Availability of PPE and Nursing Staff Ratios.

Clinical considerations	CGM in patients with COVID-19
Individualize CGM indication	• Prioritize patients with high risk of hypoglycemia • Multiple daily injections • Frail patients or those with poor nutrition, renal failure, or advanced age • High glycemic variability • Steroid-induced hyperglycemia, enteral/parenteral-induced hyperglycemia • Type 1 diabetes • Actively engaged patients for FreeStyle Libre
ICU patients on intravenous insulin infusion	• Consider transition to SC injections in patients with low insulin dose requirements (<1-2 units/kg/h).^28^ Use CGM to monitor glucose levels remotely, confirm readings with POC testing for changes in clinical condition (see Table 2). Avoid transition in patients receiving MNT, on high doses of steroids, or on vasoactive agents.
Diabetic ketoacidosis (protocols for SC insulin use evolving for mild/mod DKA)	• Consider SC protocols as opposed to IV insulin in patients with mild to moderate DKA (BG > 250 mg/dL AND acidosis AND elevated ketones), such as dose meeting the following criteria: ○ Blood gas (venous or arterial) pH ≥ 7.0 ○ Serum bicarbonate ≥ 10 mEq/L ○ Alert/Awake mental status ○ MAP > 65 after 1 L IV fluids ○ *K* ≥ 3.3 mEq/L • CGM limitations are upper glucose detection range (400-500 mg/dL) and unknown accuracy in metabolic acidosis.

Abbreviations: CGM, continuous glucose monitoring; COVID-19, coronavirus disease 2019; DKA, diabetic ketoacidosis; ICU, intensive care unit; MAP, mean arterial pressure; MNT, medical nutrition therapy (parenteral or enteral nutrition); POC, point of care; PPE, personal protective equipment; SC, subcutaneous.

Protocols for SC insulin use in patients with mild/mod DKA are evolving. A hybrid method (POC and CGM may provide appropriate guidance in selected patients) may be helpful in patients in intermediate care settings. May download Montefiore or other DKA protocol samples or protocols for transition to SC insulin at: www.covidindiabetes.org.

In patients requiring intravenous insulin therapy, based on anecdotal data, POC + CGM could aid in the care of patients requiring hourly POC testing. CGM could relieve the need for some of the frequent fingerstick blood glucose monitoring when it is adequately reflective of the POC data. For example, if a CGM reading indicates that there has not been more than a predefined small change in the past hour, then an HCW could skip an additional POC glucose test. Thus, CGM has the potential to be used to supplant POC testing in some patients, but a systematic evaluation of this approach is needed. Per a recent press release from Abbott, the Libre “is not for use on dehydrated, hypotensive, in shock, hyperglycemic-hyperosmolar state, with or without ketosis, neonates, critically-ill patients, or for diagnosis or screening of diabetes.”^5^ Similarly, Dexcom recommends “hospitals should continue to use their existing protocols to manage and treat patient glucose levels. Real-time CGM can be used to provide remote monitoring and glucose trends to aid in glucose management and medical decision making.”^4^ Health Canada authorized temporarily the use of Freestyle libre and G6 in the hospital and expanded the use of G6 to critically ill patients during COVID-19.^29^ Systematic data collection of experiences with CGM in the ICU during this pandemic is strongly encouraged.

### How to Implement CGM into the Hospital?

The implementation of diabetes technology in the hospital is rapidly evolving.^6^ Investigators from the Baltimore VA Center/University of Maryland and Emory University are together systematically evaluating the implementation of a GTS, designed to alert nursing staff to downward trending glucose values and simultaneously provide remote glucometric data to guide glycemic control. The system can be implemented with three components: a CGM device, a smartphone (a phone with internet connectivity), and a tablet (i.e., iPad). Using commercially available software applications, glucose values are sent through Bluetooth from the CGM transmitter to the smartphone, located next to the patient, and from there wirelessly to the tablet (or any other device with internet connection). This device (i.e., iPad) can be located next to the clinician/nurse or at the nursing station, and serves as a monitoring device presenting real-time CGM glucose values. With a protocol in place, staff can, by following specific instructions (i.e., hypoglycemia or hyperglycemia alerts), immediately react to correct extreme glucose values or significant trends (Tables 1 and 3). Furthermore, providers can download daily reports, using each CGM’s proprietary software, to review glycemic patterns and adjust therapy as needed (Table 3). Other software and platforms for data review, including those from open-source communities (i.e., The Nightscout Project, http://www.nightscout.info/), continue to develop to provide comprehensive real-time review of diabetes data (including CGM values, insulin doses, and carbohydrate intake), which would be helpful in the inpatient setting. However, the logistics surrounding software implementation, data security, and lack of FDA approval create barriers to its use in the hospital setting at this time.

**Table 3.** Steps for Practical Implementation.

1. Training staff	• *Benefits: Ease of use* • Simplified protocol for training: ○ Understanding alerts (setting alerts at 85 and 250 mg/dL)^30^ ○ Use of remote training program provided by companies or by institutional staff with expertise (see online material for training modules by Dexcom and Abbott)*
2. Patient selection	• Noncritically ill patients able to report glucose levels to clinical staff • Avoid patients with skin infections, anasarca, using vasoactive agents, or taking interfering substances (see above)
3. Inserting the device	• Keep devices and readers at nursing station • Swab skin with alcohol wipe • Attach CGM (back of the upper arm or abdomen, per manufacturer’s instructions) • Anticipated adhesion issues: consider adding Tegaderm or use Skin-Tac
4. Starting sensor and data sharing	• Check with receiver or smartphone to ensure CGM is operational • Abbott FreeStyle Libre: Place reader/device app near the sensor, following instructions on-screen (FreeStyle LibreLink app) • Dexcom G6: Pair device app, new transmitter and sensor, following instruction on screen (Dexcom G6 app) • Send e-mail to start sharing data with cloud-based data system (i.e., Dexcom Follow app or LibreLinkUp app)
5. Daily use and documentation	• Discuss with information technology (IT), if feasible, to build a category to manually integrate CGM readings into the electronic medical record (EMR) • For FreeStyle Libre, patients or clinical staff need to scan the sensor at least q8 hours by placing the reader/smartphone near sensor and click “check glucose” • Check a POC glucose for sensor readings <85 mg/dL or if alarms go off • For reviewing real-time data, the preference should be to use the Dexcom Follow app or the LibreLinkUp app available for smartphones • Use CGM readings to make DAILY insulin adjustments (i.e., basal-bolus) • Avoid insulin stacking by NOT making frequent changes based on arrow trends through the day • If glucose <85 mg/dL and trend arrows trending down, consider implementing hypoglycemia treatment measures in the setting of euglycemia, aiming to prevent hypoglycemia
6. Removal for procedures	• Consider placing a lead apron over the sensor for X-rays, angiogram, or fluoroscopy • Remove sensor for MRI and place new sensor after completion • No need to remove for ultrasound imaging
7. Downloading and reviewing sensor data	FreeStyle Libre: 1. Go to https://www.libreview.com/ Login Click Invite Patient Enter name, date of birth (DOB), and e-mail address, click SAVE 2. Log in the patient e-mail from a desktop or smartphone and look for Libreview invitation Accept invitation 3. CGM data are visible on LIBREVIEW to generate reports Dexcom G6: 1. Go to https://clarity.dexcom.com Click on Dexcom Clarity for Clinics Click on Add New Patient Enter Name, DOB, and click SAVE On next screen click on Share Data Select e-mail invitation Enter e-mail address and click Invite 2. Log in the patient e-mail (from either phone or desktop) and look for Dexcom Clarity invitation Copy sharing code 3. Go to patient’s phone Clarity App Paste Code Enter DOB (Year first) Accept and follow prompts 4. CGM data are visible on Dexcom Clarity to generate reports 5. Create daily report during daily rounds, and scan it into the EMR (if feasible)

Abbreviations: CGM, continuous glucose monitoring; DOB, date of birth; EMR, electronic medical record; IT, information technology; MRI, magnetic resonance imaging; POC, point of care.

* Additional information and links can be accessed at www.covidindiabetes.org

**Figure 1.**
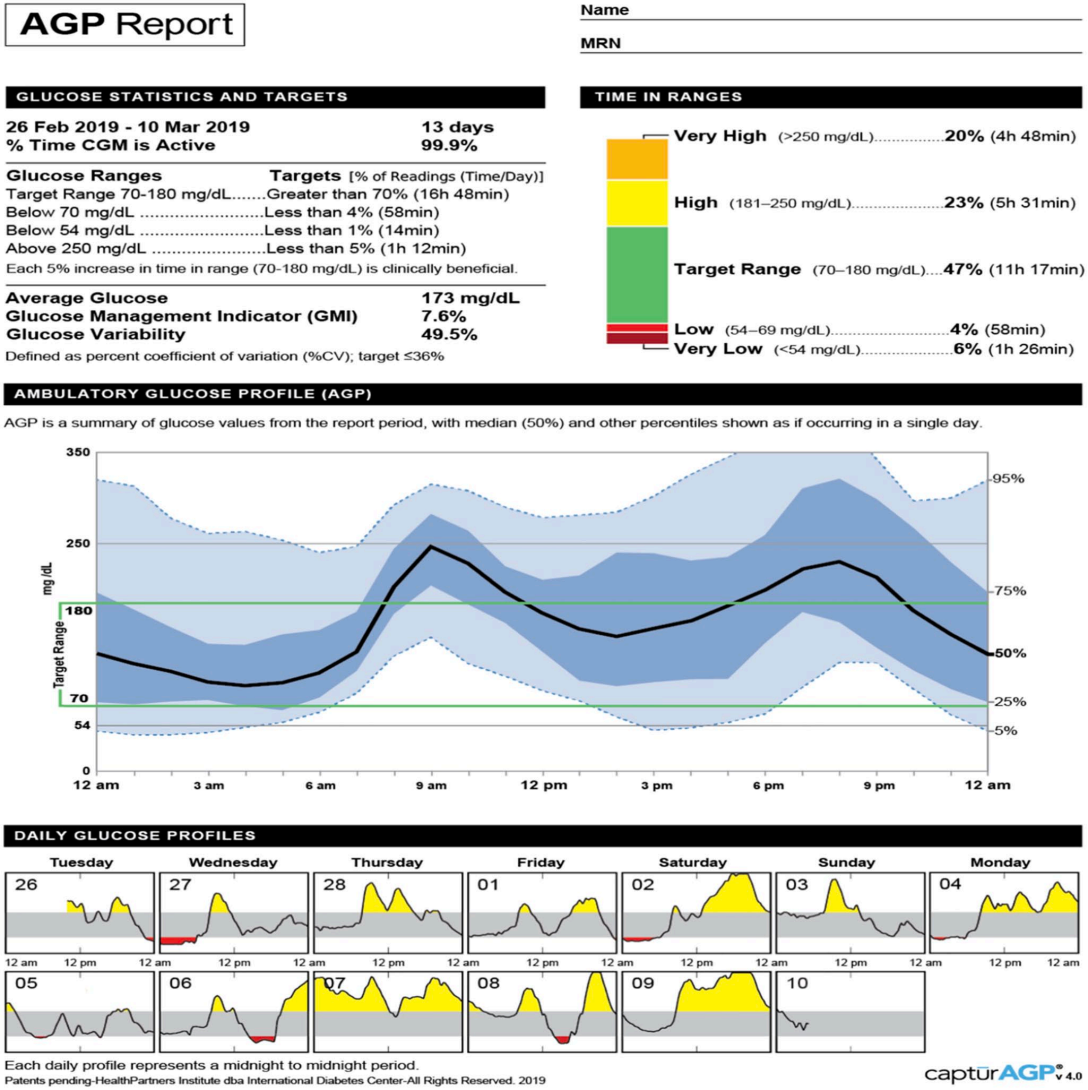
The Ambulatory Glucose Profile Report (AGP) is a standardized, single page report used in the outpatient setting.^31^ This report could be adapted for inpatient diabetes care with integration to electronic health records. Step-by-step instructions for interpreting the data: http://www.agpreport.org/agp/sites/default/files/CGM_Clinical_Guide_AGP.pdf. Source: http://www.agpreport.org/agp/about.

In the current scenario, where hospitals are emergently trying to use CGM to monitor patients remotely, the same principles apply. Using apps such as the Dexcom G6 Follow app or the Abbott FreeStyle LibreLinkUp app, providers can remotely (i.e., outside of the patient’s room) follow real-time glucose levels in the hospital and remotely. Use of the FreeStyle Libre would require the active participation of the patient or staff to periodically scan (“flash”) the sensor at a minimum of one time every eight hours (Table 1). Consider that transmission of patient CGM data to both the individual provider smartphone and a GTS may help prevent gaps in CGM data review or alert notifications in the setting of high patient care demands. Providers and hospital leadership need to become familiar with: (1) use of the equipment, including where to place the device or monitor; (2) available apps for both the patient (for their specific device) and providers (“followers”); (3) available websites for downloading and interpreting the Ambulatory Glucose Profile (Figure 1); (4) a plan and designated official to stock, purchase, store, and resupply equipment; and (5) interpretation of CGM glucose values.

Early experience from Montefiore Medical Center in New York City, which is the US epicenter of this pandemic, has indicated that staff education/training prior to implementation is key. Most HCW are unfamiliar with this technology and allowing them to look at the physical device and understand the capability of these devices has empowered them to be part of the implementation process. The staff caring for patients with COVID-19 can also help guide the selection of patients for CGM use. Consultation with diabetes hospital management team or endocrinology is frequently needed for the implementation of CGM in the hospital.

## Conclusions

The emergent need to transition to CGM to care for patients with diabetes and COVID-19 under extreme conditions has revealed the impracticality of current hospital glucose monitoring strategies. We believe that CGM may be on the threshold of becoming a widely accepted form of continuous automated physiologic monitoring in the hospital setting (depending on what future research data will show), and during the pandemic, this technology can be used to immediately address emergent needs when there is a high demand for both nursing staff and PPE. The accommodations made in the short term enabling immediate CGM use for patients with COVID-19 create an opportunity to evaluate this technology in the inpatient setting. FDA clearance will be required to move this technology from the research setting into mainstream hospital practice. Much data is needed to assess the performance of CGM in widespread hospital use. The current short-term implementation for treating COVID-19 patients during the pandemic will not generate sufficient data for assurance that CGM will be safe and effective for widespread hospital use after the pandemic. The electronic health record must be modified to accept CGM glucose data directly and economic analyses will also be needed to justify a wider implementation of this technology, but should not delay immediate implementation efforts. Today it is possible to remotely monitor cardiac rhythm and vital signs continuously on the hospital wards, where close nursing observation is not always possible.^32-34^ Given the importance of treating diabetes and uncontrolled hyperglycemia in every unit of the hospital, and the improvements in performance of current-generation CGM systems, we say why not continuously measure glucose in the hospital as well? The appropriate implementation of this technology may significantly decrease the burden of glucose monitoring for patients and providers. A systematic analysis of the experience gained during these unprecedented times will likely help transform inpatient diabetes care for the better.
